# Suspect and Target Screening of Natural Toxins in the Ter River Catchment Area in NE Spain and Prioritisation by Their Toxicity

**DOI:** 10.3390/toxins12120752

**Published:** 2020-11-28

**Authors:** Massimo Picardo, Oscar Núñez, Marinella Farré

**Affiliations:** 1 Department of Environmental Chemistry, IDAEA-CSIC, 08034 Barcelona, Spain; masqam@idaea.csic.es; 2 Department of Chemical Engineering and Analytical Chemistry, University of Barcelona, 08034 Barcelona, Spain; oscar.nunez@ub.edu; 3 Serra Húnter Professor, Generalitat de Catalunya, 08034 Barcelona, Spain

**Keywords:** natural toxins, cyanotoxins, phytotoxins, mycotoxins, suspected screening, HRMS

## Abstract

This study presents the application of a suspect screening approach to screen a wide range of natural toxins, including mycotoxins, bacterial toxins, and plant toxins, in surface waters. The method is based on a generic solid-phase extraction procedure, using three sorbent phases in two cartridges that are connected in series, hence covering a wide range of polarities, followed by liquid chromatography coupled to high-resolution mass spectrometry. The acquisition was performed in the full-scan and data-dependent modes while working under positive and negative ionisation conditions. This method was applied in order to assess the natural toxins in the Ter River water reservoirs, which are used to produce drinking water for Barcelona city (Spain). The study was carried out during a period of seven months, covering the expected prior, during, and post-peak blooming periods of the natural toxins. Fifty-three (53) compounds were tentatively identified, and nine of these were confirmed and quantified. Phytotoxins were identified as the most frequent group of natural toxins in the water, particularly the alkaloids group. Finally, the toxins identified to levels 2 and 1 were prioritised according to their bioaccumulation factor, biodegradability, frequency of detection, and toxicity. This screening and prioritisation approach resulted in different natural toxins that should be further assessed for their ecotoxicological effects and considered in future studies.

## 1. Introduction

Natural toxins in the aquatic ecosystem can be produced by different organisms, including bacteria, plants and fungi, thus grouping together a wide variety of structures and physicochemical properties and effects [1]. The risk of water contamination by natural toxins generates environmental and public health issues. In some cases, natural toxins can be accumulated in aquatic organisms and transferred throughout the aquatic food chain to humans [2].

However, if we consider freshwater environments, the primary route of human exposure includes the consumption of contaminated water, dermal exposure, and inhalation during recreational activities. Intoxication can include different symptoms, such as a severe headache, a fever, and respiratory paralysis, as well as a variety of possible effects that include hepatotoxicity, neurotoxicity, carcinogenicity, and dermal toxicity. Due to their diversity, toxicological assessment is still challenging and there is also an information gap concerning their occurrence, due to the lack of analytical methods and certified standards. Therefore, the concentration of natural toxins in drinking water for most of these groups is not yet well regulated, and this is also of concern for countries in the European Union (EU).

Among the natural toxins, the cyanotoxins group is one of the most studied groups in freshwater ecosystems. Cyanotoxins can be released by cyanobacterial blooms, which is a frequent natural phenomenon that is characterised by an algal biomass accumulation in surface water. These secondary metabolites include hepatotoxins (microcystins and nodularins), neurotoxins (such as anatoxins, saxitoxins, and β-methylamino-l-alanine), cytotoxins (such as cylindrospermopsin), and dermatotoxins (lipopolysaccharide, lyngbyatoxins, and aplysiatoxin). Among them, microcystins (MCs), produced by freshwater cyanobacteria genera such as *Microcystis, Aphanizomenon, Planktothrix, Dolichospermum*, etc. [3], are the most diverse group and the best described in the literature [4]. However, only one congener is regulated. The World Health Organization (WHO) has issued a guideline value of 1 µg/L in drinking water for microcystin-LR (MC-LR), which is one of the most toxic and widespread toxins in water supplies [5].

Another relevant group is represented by mycotoxins, which are secondary metabolites produced by fungi. Due to their diverse chemical structures, mycotoxins can present a wide range of toxicity, such as hepatotoxicity, nephrotoxicity, neurotoxicity, and immunotoxicity, and some of them have been recognised as being teratogenic, mutagenic, and carcinogenic [3]. Their biological effects have been extensively reported and regulated in food and feed [6,7] but not in water. However, many environmental species (particularly of the genus *Aspergillus*) show resistance to the commonly used water disinfection procedures, allowing them to enter water distribution/reticulation systems [8,9]. Moreover, those species can form mixed biofilm communities with bacteria, algae, and protozoa. These biofilms increase the ability to survive heat treatments and chlorination procedures. Therefore, fungal presence in tap water distribution systems also leads to an increase in the presence of temperature-tolerant fungi, which are the target of many studies that note this as a serious health risk [10].

The phytotoxins group includes secondary metabolites that are produced by plants as a defence mechanism against herbivores, insects, or other plant species [11]. They can include different chemical structures, including peptides, terpenoids, flavones, glycosides, and phenolic compounds (<3500 Da) [12]. Phytotoxins can be grouped into three major chemical structures: alkaloids, terpenes, and phenols. Among them, furanocoumarins, lectins, glycoalkaloids, and pyrrolizidine alkaloids are the most studied [1,13,14]. These compounds can end up in water bodies due to leaching from leaves and soil, and some of them can present high toxicity, such as the case of the carcinogenic ptaquiloside, which is produced by bracken fern [15]. However, in general, few studies have explored their presence in surface waters [16], despite their potentially high toxicity alone or in combination with other anthropogenic contaminants.

During the recent decades, the contamination and over-enrichment of nutrients (eutrophication) of surface waters have increased the number of harmful algal bloom events. Moreover, the increasing temperatures and light intensity promote the algal bloom events and consequently the production of natural toxins [17]. Their chemical diversity, the variety of their structures with structural features that are comparable to common anthropogenic contaminants, and their low concentrations can lead to harmful effects, making their determination in surface waters a great challenge. For these reasons, it is of primary importance to investigate the occurrence of natural toxins in the aquatic environment.

The most common approaches using multi-residue analysis include a limited number of compounds [18,19]. Most approaches cannot determine a wide range of polarities, in that they are mostly applied for one particular compound or a group of compounds with similar characteristics. The suspect screening methods that are based on high-resolution mass spectrometry (HRMS) opened a new window for the comprehensive study of natural toxins in surface waters.

In this regard, the main goal of the present study was to apply a recently developed method [20], based on a generic three-step solid-phase extraction (SPE) procedure followed by liquid chromatography (LC) coupled to high-resolution mass spectrometry (HRMS), with full-scan (FS) and data-dependent MS^2^ (DDA) acquisition using a Q-Exactive Orbitrap analyser, to study the natural toxins in different water reservoirs that are used to produce tap water in Barcelona city (Catalonia, NE Spain).

Here, we present the data that was originated by the analysis of a complete set of samples that were collected during a sampling campaign in the period of March to September 2018. The data reported in the previous work have been omitted in the present one. In this sampling campaign, the 48 samples were collected at 4 sites along the Ter River. Sample collection was carried out twice a month from March to September 2018. In our previous study, the 16 samples that came from the Ter River were collected using a different sampling campaign, specifically designed to assess the good performance of the newly developed approach, and was carried out in May and July, and thus needless to say at different days from the samples presented here. Moreover, a prioritisation protocol, including a scoring system, is reported now, designed to elucidate the most significant natural toxins of concern in the drinking water reservoirs.

The suspect screening was carried out using a suspect list containing 2384 items of natural toxin data that were collected from the literature and online databases (mzCloud and ChemSpider). The confidence levels for the identification of suspect natural toxins were based on the approach that was previously reported by Sckymansky et al. [21], consisting of mass accuracy, isotopic fit, fragmentation, and final confirmation, using standards and retention times. Finally, the suspect natural toxins were prioritised according to their toxicity, frequency of detection, biodegradability, and bioaccumulation factors. The results of this screening and prioritisation protocol present a set of natural toxins that could be assessed for their toxicological effects and should also be considered in future water monitoring studies. To the best of our knowledge, this is the first study providing the prioritisation of natural toxins in a water reservoir in Spain.

## 2. Results and Discussion

### 2.1. Tentatively Identified Compounds

**Figure 1:**
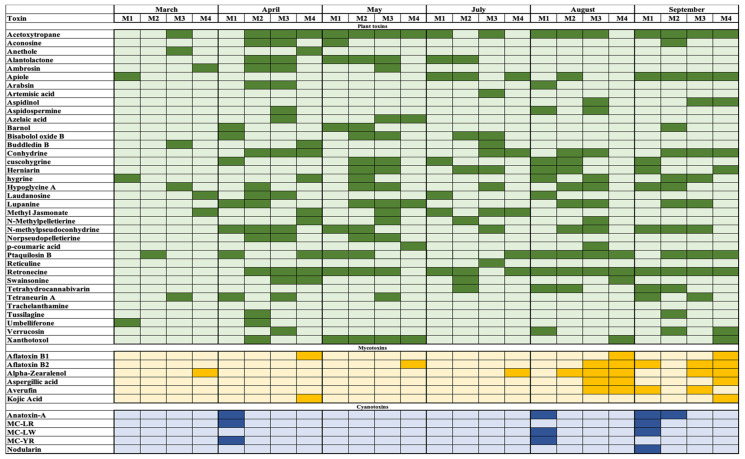
Hits diagram. A dark colour indicates a positive hit.

In this study, after removal of the background and the very small signals under the minimum intensity threshold, 4404 suspect masses were detected in the 48 water samples by using Compound Discoverer 3.1 software. Among them, 381 compounds (8.6%) were assessed as suspect natural toxins that were included in the in-house database and finally selected for further screening. It is noteworthy that the compounds of the study were natural toxins pertaining to three major groups in water, phytotoxins, mycotoxins, and cyanotoxins. Other compounds, such as pesticides, were discarded in this study. Among these 381 structures, after filtering by way of the isotopic patterns, ionisation efficiency, and fragmentation patterns, the number of suspected identified compounds diminished to 191 structures (50.1% of the initial potential for natural toxins). Finally, the comparison with in-silico MS^2^ patterns gave 50 structures that were tentatively identified at level 2 (25.7% of the initial potential for natural toxins) (Table 1 and Figure 1). Finally, nine natural toxins were confirmed and quantified by injections of the standard.

**Figure 2:**
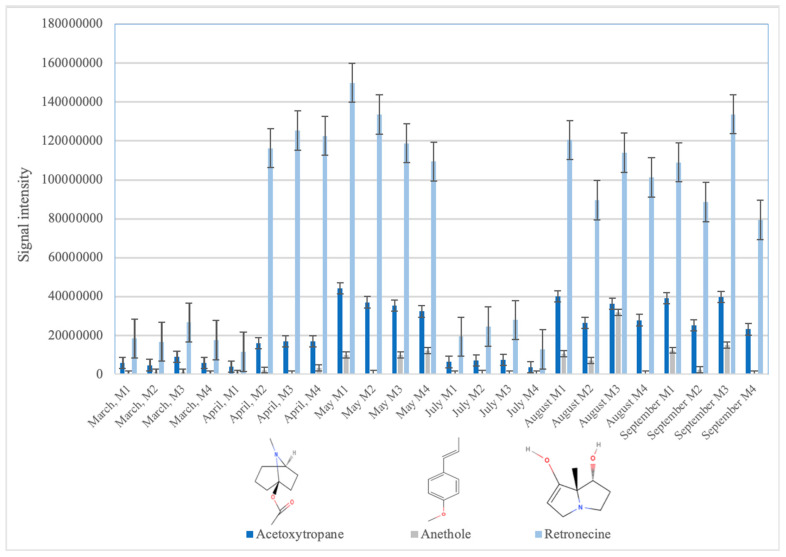
Signal intensities of three alkaloids: acetoxytropane, anethole, and retronecine.

**Figure 3:**
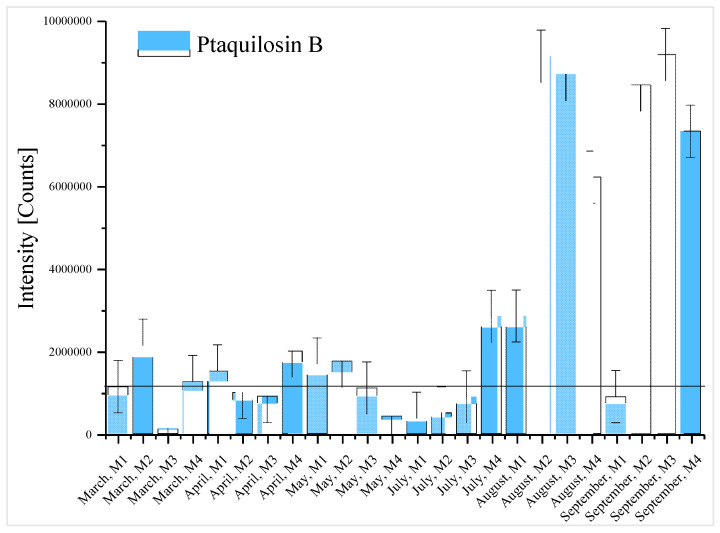
Ptaquilosin B intensity signals along the sampling period.

Plant toxins were the most prominent group in the studied samples (73% of the tentatively identified compounds), with a prevalence of the alkaloids group. The most frequently identified phytotoxins were acetoxytropane, retronecine, and N-methyl pseudo conhydrine in 71%, 70%, and 46% of the samples, respectively. These results are in agreement with the diversity of endemic plants of the area [22], due to the different climatic zones of the occidental Pyrenees and the variation in dry and wet periods. The occurrence of some of these toxins was at a maximum in April, May, August, and September. These two peaks of natural toxins can be related to the leaching into the water immediately after the flowering period in the Mediterranean area, corresponding to April and May, and posteriorly the release of toxins from the dead plant with the consequent rain-washing effect into the river in August and September. For example, in Figure 2, the intensity of the signals of three alkaloids, acetoxytropane, anethole, and retronecine, which can be attributed to the *Symphytum officinale*, *Pimpinella anisum* [23], and *Apiaceae* families, are displayed. As can be seen, the maximum intensities of the toxins were between May and September. In addition to the alkaloids, some terpenes were also tentatively identified. A common species in this area and in the general region of the Iberian Peninsula is bracken (*Pteridium aquilinum*) [24], which produces ptaquiloside [15]. Ptaquiloside is a carcinogen norsesquiterpene glucoside that is responsible for haemorrhagic disease and bright blindness in livestock and can produce gastric cancer in humans [25]. As can be seen in Figure 1, in this study the degradation product of ptaquiloside, ptaquilosin B (PTB) [26], was identified in 33% of the samples, while ptaquiloside was not detected. The degradation of ptaquiloside in soils and the start of the rainy season explains the leaching of PTB into the water, which is coincident with the maximum intensities of the signals in the samples that were collected in August and September (Figure 3). Another relevant group of phytotoxins, the phenolic group, was less represented in the samples that were identified, and the representatives of this group were present in a minor number of samples. An example was p-coumaric acid, which was found in only 8% of the samples.

**Figure 4:**
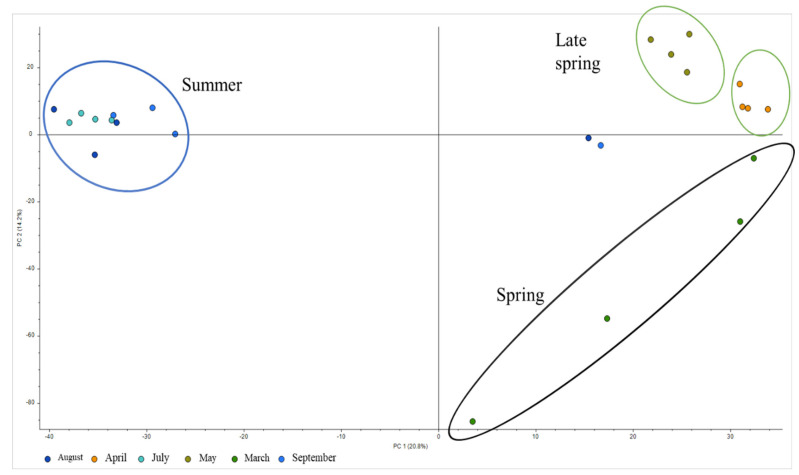
PCA of the results during the sampling period.

Mycotoxins were marginally detectable in the samples, and 58% of the studied water samples did not present detectable concentrations. Alpha-zearalenol was the most prevalent suspect mycotoxin with an occurrence of 29%, followed by aflatoxin B_2_ (25%), aflatoxin B_1_ (12%), and averufin, which is an anthraquinoid precursor of aflatoxins [27,28]. Regarding the distribution during the study period, mycotoxins were almost exclusively detected in August and September when the rainy season started, indicating that their presence in water could be due to the washing effect of plants infected with *Aspergillus flavus* and *Aspergillus parasiticus* in the case of aflatoxins and *Fusarium* mycotoxins in the case of alpha-zearalenol. As can be seen in Figure 2, and on the principal component analysis (PCA) presented in Figure 4, the occurrence of natural toxins in natural waters is influenced by seasonality, and the months with a higher charge of natural toxins were in this case April, August, and September, while a very low presence of natural toxins was found at the end of winter and during the driest months. Contrary to what can be expected, the samples from May and July were almost free of cyanotoxins. Only in M1 and M2 during April, August, and September was the occurrence of cyanotoxins detected, in agreement with the two peaking algal blooms in the Mediterranean region. This site (M1) corresponded to the area of Pasteral dam, which is the reservoir that is located downstream of the other reservoirs and presenting slightly higher levels of eutrophication in comparison with the other three areas. The more frequently found cyanotoxins were anatoxin-a, which was present in four samples, followed by microcystin LR, LW, and YR.

The concomitant presence of three MCs, both with anatoxin-a, at the sampling point M1, suggests this area is of a higher risk in terms of the occurrence of MCs, and therefore of MC producers. This is in line with the previous studies reporting benthonic species in the NE of Catalonia. Thirty-two different species have been identified as endemic in this area [29]. Toxins producing genera of freshwater cyanobacteria include *Phormidium* spp., *Oscillatoria* spp., *Nostoc* spp., and *Pseudanabaena* spp. [27]. These were considered to be the main producers of MC-LR, MC-YR, and –LW found in the M1 point in May and July. The occurrence of cyanotoxins can be related to increments in temperature and eutrophication, as was confirmed by the Catalan Water Agency [28] and CARIMED 2018 [30] for this area during the period studied. On the other hand, M1 is the downstream point of the studied area, which receives nutrients from areas in the upper river, with nitrate levels between 0.67 and 10 mg N-NO_3_^−^/L.

### 2.2. Target Analysis

A target analysis of 27 natural toxins was carried out using certified standards that are summarised in Table A1 of Appendix A. Matrix-matched calibration curves were used for the quantification of eight natural toxins. The limits of detection (LODs) were between 0.002 to 0.4 µg/L while the limits of quantification (LOQs) were between 0.07 and 1.5 µg/L. The analytical parameters are summarised in Table A3. Nine toxins were confirmed (Ana, AflB1, MC-LR, MC-LW, Nod, MC-YR, Kja, 7-methoxycoumarin, and umbelliferone). Concentrations were under the limit of 1 µg/L as proposed by the World Health Organisation [24] and they were used as an arbitrary reference limit in this work. MC-LR was confirmed in only two sampling points (April M1 and September M1), where the precursor ion [M + H]^+^ 995.5560 *m/z* was detected for both with the fragment 135.0806 *m/z*, which is typically generated by the ADDA structure. Finally, MC-LR was confirmed with standards in these two samples. MC-LW and MC-YR were detected at the M1 point in September, August, and, surprisingly, in April, which correspond to the same months where the MC-LR was detected. Anatoxin-a was further detected in the same periods. 7-methoxycoumarin and umbelliferone were confirmed by certified standards. The concentrations of the detected natural toxins are reported in Table 2, showing their presence at relatively low levels in water.

### 2.3. Prioritisation

In this study, a scoring system was designed to highlight the most significant natural toxins of concern in drinking water reservoirs. The scoring system was in accordance with the previous protocol that was published by Choi et al. [31], which is based on the risk-relevant parameters such as the detection frequency in percentage, biodegradability, log BAF, and the toxicity values based on the 50% lethal dose (LD50) laboratory tests in mice. A score in the range of 0 to 100 for each parameter was used, and 100 points were additionally added if carcinogenicity or neurotoxicity was already reported for the substance as what happens, for example, with AflB_1_ and AflB_2_. Thus, the maximum total for a given toxin can be 500. In Table 3, detailed information on the parameterisation and scoring is provided, and in Table 4, the parameters used for each tentatively identified substance are shown. It is noteworthy that the biodegradability and the bioaccumulation factor (BAF), used as log BAF, were calculated using EPI Suite^TM^ software (United States Environmental Protection Agency, U.S. EPA).

In Table 5, the ranking of the tentatively identified substances is presented. Four substances, namely, tetrahydrocannabivarin, MC-LW, aconosine, and MC-LR, were ranked with more than 300 points, and 13 toxins were ranked with more than 200 points. In this case, it was considered to be the frequency during the sampling period, which includes seasons with a lower incidence of the substances in water.

However, following a month-by-month inspection, for certain substances the frequency was higher; hence, this ranking then varies a little and a higher number of toxins reaches 300 points.

For this reason, in spite of the low concentrations of the substances that are quantified as the top 12 toxins to be tentatively identified, Barcelona city water reservoirs should be monitored at least from May to September, which were the months with higher occurrences of natural toxins.

## 3. Conclusions

The method described in this article is a good alternative for tentatively identifying suspect natural toxins in surface water. We have shown that the presence of organic matter near the river can potentially cause the leaching of mycotoxins. Moreover, in this study, plant toxins were mostly spread across different points in relation to the presence of different endemic plants. Notwithstanding, the botanical diversity influences the presence of natural toxins as equally as the precipitation and dry periods. The concentrations of natural toxins were not determined due to the lack of certified standards; however, a correlation between the rain and the leaching in water was described and assessed.

Thanks to these results, we report on the importance of the suspect screening for the identification of natural toxins and their final inclusion in prioritisation lists in order to control their presence in water environments, in particular in drinking water reservoirs. It is also important to increase the amount of data, to help scientists identify environmental compounds when no standards are available, or where they are excessively expensive. Many MC congeners are still not included in databases such as MzCloud and Chemspider. Hence, the retrieval of MS^2^ spectrums for the MC congeners is an issue that is being solved with the efforts of the scientific community via the constant updating of data in dedicated databases for environmental research. For comparison purposes, future works should apply this method of analysing natural toxins across different climates worldwide.

## 4. Materials and Methods

### 4.1. Chemicals and Reagents

Twenty-seven (27) natural toxin standards with a maximum purity between 95 and 99% were selected for the targeted analysis. In Table A1 of Appendix A, the list of standards, their main chemical parameters, and providers are listed. Methanol (MeOH), acetone, and acetonitrile (ACN) of HPLC grade were from Merck (Darmstadt, Germany). HPLC water grade was from Baker (Madrid, Spain).

### 4.2. Samples and Sampling Sites

Forty-eight surface water samples were collected from the Ter River (Catalonia, NE Spain) at four sampling sites: (M1) 41.986133, 2.603488; Point 2 (M2) 41.982191, 2.585539; Point 3 (M3) 41.991090, 2.570144; and Point 4 (M4) 41.975693, 2.395398, in the area of Pasteral, Susqueda, and Sau dams, which are the freshwater reservoirs for Barcelona city tap water.

The sampling was carried out from March to September 2018, except for June, twice per month, in order to study the prior, during, and after blooming periods, when higher concentrations of natural toxins are expected [77]. In each sampling site, the pH, conductivity, and pO_2_ were measured. Water samples were collected in amber glass bottles that had previously been rinsed, transported at 4 °C, and maintained frozen at −40 °C until the start of the analytical process.

### 4.3. Sample Pre-Treatment

Sample pre-treatment was based on the generic methodology to isolate natural toxins from water, as recently developed by Picardo et al. [20]. Briefly, each sample was processed in an ultrasonic bath for 20 min to disrupt the microbial cells and to release the intracellular toxins. Then, the sonicated samples were filtered through a glass microfibre filter of GF/B grade (Sigma Aldrich, Steinheim, Germany). Natural toxins were isolated from the filtrate via a three-step solid-phase extraction (SPE) method, using a hand-made cartridge that had been prepared with 200 mg of a porous graphitised carbon (PGC) 120 mesh (Sigma Aldrich, Steinheim, Germany) and 200 mg of a Bond-Elut PPL (PPL) 120 mesh (Agilent, Santa Clara, CA, USA), coupled to an HLB plus cartridge (225 mg sorbent) (Waters Corporations, Milford, MA, USA).

Then, water samples, each of 100 mL, were loaded into the cartridges at a flow rate of 2 mL/min, previously conditioned with 10 mL of MeOH and 10 mL of water, and both solvents were acidified with 0.5% of formic acid (FA). After loading, the cartridges were dried and switched to elute the analytes in the backflush mode. The PGC/PPL cartridge was reversed, while the HLB cartridge maintained the same position. The toxins were eluted with 15 mL of water/MeOH 2:8 (*v/v*), followed by 15 mL of MeOH and 15 mL of acetone/MeOH 50:50 (*v/v*). All the solvents were previously warmed at 45 °C before each elution. The eluate was evaporated almost to dryness and re-dissolved in 1 mL of the mobile phase.

### 4.4. Liquid Chromatography Coupled with High-Resolution Mass Spectrometry

According to the method described by Picardo et al., 2020 [20], the chromatographic separation was carried out using a C18 reversed-phase Lichrosphere (125 mm × 2 mm i.d., 5 μm) column (Merck, Barcelona, ES) connected to an Acquity high-performance liquid chromatography system (Waters Corp, Milford, MA, USA). The binary mobile phase was composed of water (solvent A) and acetonitrile (solvent B) and both had been acidified with 0.1% of FA. The elution gradient was as follows: from 0–3 min, 10% B; from 3–13 min, B was linearly increased to 90%; 13–15 min, stabilised at 90% B; 15–16 min B decreased linearly to 10%; 16–20 min, column stabilisation with 10% of solvent B. A 20 μL injection volume was used with a mobile phase flow rate of 0.25 mL/min.

The HPLC system was coupled to a Thermo Scientific Orbitrap Q-Exactive mass spectrometer (Thermo Fisher Scientific, San Jose, CA, USA) equipped with a heated electrospray ionisation source (HESI), and used in the positive and negative ionisation modes. The acquisition was performed using a full-scan and data-dependent analysis (FS-DDA) from *m/z* = 75 to *m/z* = 1100, with a resolution of 35,000 full widths at half maximum (FWHM) for the FS and 17,500 FWHM for the DDA There was a spray voltage of 3.75 kV (+) and −3.25 kV (−), a sheath flow gas of 20 a.u., an auxiliary gas of 20 a.u., and a sweep gas of 5 a.u. Heater and capillary temperatures were set at 300 °C with an S-lens RF level at 60%. An inclusion list of the 100 most probable suspect compounds was used (Appendix A Table A2).

### 4.5. Data Processing: Suspect Screening of Natural Toxins

**Figure 5:**
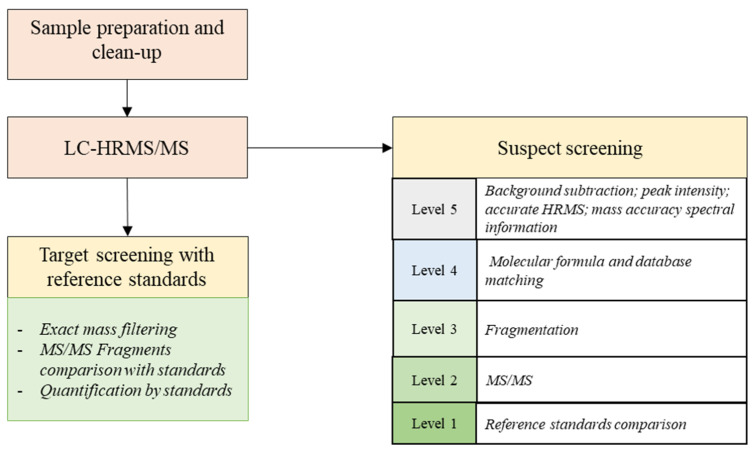
General workflow for suspect screening as proposed by Schymansky et al. [21].

The suspect screening procedure that was previously described by Picardo et al. [20] was employed with minor changes. Briefly, the FS chromatograms that were obtained with the acquisition software Xcalibur Qual Browser (Thermo Fisher Scientific) were processed, using an automated screening with Compound Discoverer software version 3.1 v. x86 (Thermo Fisher Scientific, San Jose, CA, USA). The first screening steps included peak picking, RT alignment, and grouping of isotopes and adducts (to form compounds), as well as the grouping of compounds across samples. Suspect compounds were marked as background if their peak area in the samples was less than three times larger than the maximum peak area in the blanks. Suspects were tentatively identified using the exact mass with a mass error of 5 ppm. This created a first list of suspect compounds that were further filtered by comparison with a homemade database containing the exact mass of more than 2384 natural toxins. Further filtering steps consisted of the comparison of isotopic patterns, ionisation efficiency, and fragmentation patterns. In Figure 5, the general workflow is summarised, which is similar to the workflows of Krauss [78] and Schymanski [21]. Finally, the MS/MS spectrum was compared with the spectrum of a standard or the predicted fragmentation pattern using the ChemSpider and MzCloud online databases. Unequivocal confirmation was only possible when a reference standard was available (identification at level 1).

### 4.6. Accuracy, Precision, Limits of Detection, and Quantification

Quantification was achieved through calibration curves that were prepared in an artificial freshwater matrix (AFW). The AFW was prepared using the same ingredients that were reported by Lipschitz and Michel [79]. Briefly, the organic matter was simulated with 10 mg/L of technical grade humic acid (Sigma-Aldrich, reference 53,680), and the pH was adjusted to 6.5 with 1.0 M formic acid. Matrix-matched calibration curves were produced using spiked samples from 0.5 to 100 µg/L. Intra-assay precision, accuracy, LOD, and LOQ for the confirmed toxins were calculated according to the EURACHEM guidelines [80]. The instrumental limits of detection (iLOD) were obtained by progressive dilution to the lowest concentration, whereby each compound could be detected. Instrumental reproducibility (inter-day precision) was calculated as the average percentage of the relative standard deviation (RSD%) of the standard solutions (six replicates) at seven concentration levels on three consecutive days.

## Figures and Tables

**Table 1:** List of suspect compounds (level 2) after tentative identification in the four sampling sites along water reservoirs in the Ter River.

Toxins	Formula	[M + H]	Rt	MS2 (1)	[M-e]+	MS2 (2)	[M-e]+	MS2 (3)	[M-e]+	MS2 (4)	[M-e]+
Plant Toxins											
Acetoxytropane	C_10_H_17_NO_2_	184.1332	9.1	123.0805	C_8_H_11_O	142.0864	C_7_H_12_NO_2_	125.0599	C_7_H_9_O_2_	165.0913	C_10_H_13_O_2_
Aconosine	C_22_H_35_NO_4_	378.2639	11.3	283.1701	C_19_H_23_O_2_	269.1539	C_18_H_21_O_2_	235.1324	C_14_H_19_O_3_	137.0599	C_8_H_9_O_2_
Anethole	C_10_H_12_O	149.0961	9.8	115.0544	C_9_H_7_	103.0543	C_8_H_7_	145.065	C_10_H_9_O	121.0649	C_8_H_9_O
Ambrosin	C_15_H_18_O_3_	247.1332	8.5	229.1227	C_15_H_17_ O_2_	201.1267	C_13_H_13_O_2_	119.0857	C_9_H_11_		
Apiol	C_12_H_14_O_4_	223.0965	11.9	105.07	C_8_H_9_	119.0857	C_9_H_11_	163.0755	C_10_H_11_O_2_	149.0963	C_10_H_13_O
Arabsin	C_15_H_22_O_4_	266.1521	10.8	249.1488	C_15_H_21_O_3_	231.1384	C_15_H_19_O_2_	221.1539	C_14_H_21_O_2_		
Artemisic acid	C_15_H_22_O_2_	235.1702	14	179.1069	C_11_H_15_O_2_	165.0901	C_10_H_13_O_2_	119.0853	C_9_H_11_		
Aspidinol	C_12_H_16_O_4_	225.1121	9.5	107.0492	C_7_H_7_O	137.0599	C_8_H_9_O_2_	123.0441	C_7_H_7_O_2_	109.0649	C_7_H_7_O
Aspidospermine	C_22_H_30_N_2_O_2_	355.2380	12.5	107.0492	C_7_H_7_O	136.0759	C_8_H_10_NO	174.0915	C_11_H_12_NO	148.0759	C_9_H_10_NO
Azelaic acid	C_9_H_16_O_4_	189.1121	11.0	107.0854	C_8_H_11_	155.0704	C_8_H_11_O_3_	111.0806	C_7_H_11_O	115.0391	C_5_H_7_O_3_
Barnol	C_10_H_14_O_3_	183.1016	10.8	119.0857	C_9_H_11_	135.0806	C_9_H_11_O	163.0755	C_10_H_11_O_2_	181.086	C_10_H_13_O_3_
Bisabolol oxide	C_15_H_26_O_2_	239.2006	12.4	133.1013	C_10_H_13_	121.1013	C_9_H_13_	149.1326	C_11_H_17_	187.1483	C_14_H_19_
Buddledin B	C_15_H_22_O_2_	235.1693	12.9	113.0598	C_6_H_9_O_2_	179.0106	C_11_H_15_O_2_	193.1225	C_12_H_17_O_2_	155.1067	C_9_H_15_O_2_
Conhydrine	C_8_H_17_NO	144.1383	11.6	107.0856	C_8_H_11_	125.0962	C_8_H_11_O	138.0915	C_8_H_12_NO		
Cuscohygrine	C_13_H_24_N_2_O	225.1961	12.3	123.0805	C_8_H_11_O	109.0649	C_7_H_9_O	163.1118	C_11_H_15_O	150.0914	C_9_H_12_NO
Curassavine	C_16_H_29_NO_4_	300.2169	12.6	155.0703	C_8_H_11_O_3_	107.0856	C_8_H_11_	123.0805	C_8_H_11_O	173.081	C_8_H_13_O_4_
Herniarin	C_10_H_8_O_3_	176.0477	11.8	121.0649	C_7_H_5_O_2_	133.0653	C_9_H_9_O				
Hydroxyarbusculin A	C_15_H_22_O_4_	267.1585	13.3	159.1169	C_12_H_15_	123.0805	C_8_H_11_O				
Hydroxycoumarin	C_9_H_6_O_3_	163.0390	15.1	121.0284	C_7_H_5_O_2_	149.0233	C_8_H_5_O_3_	163.0389	C_9_H_7_O_3_	105.0335	C_7_H_5_O
Hygrine	C_8_H_15_NO	142.1226	10.9	109.065	C_7_H_9_O	124.0758	C_7_H_10_NO	111.0804	C_7_H_11_O	140.1069	C_8_H_14_NO
Hypoglycine A	C_7_H_11_NO_2_	142.0862	2.34	97.0287	C_5_H_5_O_2_	120.0444	C_7_H_6_NO	124.0757	C_7_H_10_NO		
Laudanosine	C_21_H_27_NO_4_	358.2013	13.2	121.0285	C_7_H_5_O_2_	115.0543	C_9_H_7_	159.088	C_11_H_11_O	147.0805	C_10_H_11_O
Lupanine	C_15_H_24_N_2_O	249.1961	5.3	110.0965	C_7_H_12_N	120.0808	C_8_H_10_N	122.0966	C_8_H_12_N	138.0915	C_8_H_12_NO
Methyl Jasmonate	C_13_ H_20_ O_3_	225.1485	0.1	107.0855	C_8_H_11_	121.1012	C_8_H_13_	175.112	C_12_H_15_O	165.1275	C_11_H_17_O
Methylpelletierine	C_9_H_17_NO	156.1386	2.2	107.0705	C_8_H_11_	140.105	C_8_H_14_N_O_				
Methylpseudoconhydrine	C_9_H_19_NO	158.1539	11.9	107.0856	C_8_H_11_	114.0914	C_6_H_12_NO	123.0805	C_8_H_11_O	109.0649	C_7_H_9_O
Norpseudopelletierine	C_8_ H_13_NO	140.1070	9.1	109.0649	C_7_H_9_O	121.0649	C_8_H_9_O	138.0917	C_8_H_12_NO	123.0806	C_8_H_11_O
p-Coumaric acid	C_9_ H_8_ O_3_	165.0546	12.5	105.07	C_8_H_9_	123.0441	C_7_H_7_O_2_	133.0649	C_9_H_9_O	125.0598	C_7_H_9_O_2_
Ptaquilosin B	C_14_ H_20_ O_3_	237.1485	11.2	119.0857	C_9_H_11_	159.0807	C_11_H_11_O	145.1013	C_11_H_13_	111.0442	C_6_H_7_O_2_
Reticuline	C_19_ H_23_ N O_4_	330.1700	13.2	115.0543	C_9_H_7_	125.0597	C_7_H_9_O_2_	145.0646	C_10_H_9_O	135.0441	C_8_H_7_O_2_
Retronecine	C_8_ H_13_ N O_2_	156.1019	1.9	152.0709	C_8_H_10_NO_2_	118.0652	C_8_H_8_N	114.0916	C_6_H_12_NO	124.0758	C_7_H_10_NO
Swainsonine	C_8_ H_15_ N O_3_	174.1125	8.1	140.0682	C_7_H_10_NO_2_	114.0914	C_6_H_12_NO	125.0598	C_7_H_9_O_2_	118.0652	C_8_H_8_N
Tetrahydrocannabivarin	C_19_ H_26_ O_2_	287.2006	12.9	105.07	C_8_H_9_	163.1118	C_11_H_15_O	175.0755	C_11_H_11_O_2_	217.0123	C_14_H_17_O_2_
Tetraneurin A	C_17_ H_22_ O_6_	323.1489	12.6	281.0996	C_14_H_17_O_6_	199.0968	C_19_H_15_O_4_	155.0704	C_8_H_11_O_3_	213.112	C_11_H_17_O_4_
Trachelanthamine	C_15_ H_27_ N O_4_	286.2013	12.5	155.0704	C_8_H_11_O_3_	107.085	C_8_H_11_	159.0655	C_7_H_11_O_4_	215.1269	C_11_H_19_O_4_
Tussilagine	C_10_ H_17_ N O_3_	200.1281	10.6	180.1021	C_10_H_14_NO_2_	165.0912	C_10_H_13_O_2_	151.0756	C_9_H_11_O_2_	134.0967	C_9_H_12_N
Umbelliferone	C_9_ H_6_ O_3_	163.0390	11.1	147.0441	C_9_H_7_O_2_	135.0442	C_8_H_7_O_2_	111.0441	C_6_H_7_O_2_	123.0441	C_7_H_7_O_2_
Verrucosin	C_20_ H_24_ O_5_	345.1697	13.0	301.143	C_18_H_21_O_4_	121.0286	C_7_H_5_O_2_	141.0548	C_7_H_9_O_3_	247.1332	C_15_H_19_O_3_
Xanthotoxol	C_11_H_6_O_4_	203.0348	1.3	147.1173	C_9_H_10_O_2_	177.0188	C_9_H_5_O_4_	173.0239	C_10_H_5_O_3_		
**Mycotoxins**											
Aflatoxin B_1_	C_17_H_12_O_6_	313.0707	11.2	213.0547	C_13_H_9_O_3_	269.0444	C_15_H_9_O_5_	285.0761	C_16_H_13_O_5_	217.0497	C_12_H_9_O_4_
Aflatoxin B_2_	C_17_ H_14_ O_6_	315.0863	11.6	273.0761	C_15_H_13_O_5_	255.0654	C_15_H_11_04	68.9979	C_3_HO_2_		
Alpha-Zearalenol	C_18_H_24_O_5_	321.1674	14.8	149.133	C_11_H_17_	121.1016	C_9_H_13_	139.1123	C_9_H_15_O		
Aspergillic acid	C_12_ H_20_ N_2_ O_2_	225.1598	9.4	114.0915	C_6_H_12_NO	144.0889	C_6_H_12_N_2_O_2_	150.0915	C_9_H_12_NO	128.07	C_6_H_10_NO_2_
Averufin	C_20_ H_16_ O_7_	369.0969	10.6	327.0853	C_18_H_15_O_6_	299.0555	C_16_H_11_O_6_	137.0236	C_7_H_5_O_3_		
Kojic Acid	C_6_H_6_O_4_	143.0344	1.38	125.0239	C_6_H_5_O_3_	97.02844	C_5_H_5_O_2_	69.0335	C_4_H_5_O		
**Cyanotoxins**											
ANA-a	C_10_H_15_NO	166.1226	0.5	149.1	C_10_H_13_O	131.0859	C_10_H_11_	107.0858	C_8_H_11_		
MC-LR	C_49_H_74_N_10_O_12_	995.556	9	135.0807	C_9_H_11_O	213.087	C_9_H_13_N_2_O_4_	375.1914	C_20_H_27_N_2_O_5_		
MC-LW	C_54_H_72_N_8_O_12_	1025.5343	12	135.0807	C_9_H_11_O	376.1926	C_19_H_21_N_10_	288.1354	C_17_H_20_O_4_		
MC-YR	C_52_H_72_N_10_O_13_	1045.5317	8.9	135.0807	C_9_H_11_O	375.1935	C_19_H_21_N_9_	213.0874	C_9_H_13_N_2_O_4_		
NOD	C_41_H_60_N_8_O_10_	824.4446	8.6	135.0807	C_9_H_11_O	389.2079	C_21_H_29_NO_5_	691.3795	C_34_H_53_O_10_N_5_		

**Table 2:** Quantification of the confirmed compounds detected in the Ter River.

Toxin	Month	Sampling Point	Concentration (µg L^−1^)
Ana-a	April	M1	0.12
	August	M1	0.03
	September	M1	0.06
	September	M2	0.28
Afla B_1_	September	M4	0.9
Kja	April	M4	0.7
Nod	September	M1	0.1
MC-YR	April August	M1 M1	0.1 0.2
MC-LW	August	M1	0.4
	September	M1	0.1
MC-LR	April	M1	0.2
	September	M1	0.7
Umbelliferone	May	M3	<LOD
	July	M2 M3	<LOD 0.1
	August	M2 M3	<LOD <LOD
7-methoxycoumarin	May	M2 M3	0.17 0.008
	July	M2 M3	0.08 0.18
	August	M2 M3	0.06 0.03
	September	M1	0.04

Abbreviations: Afla B_1_: aflatoxin B_1_; Ana-a: anatoxin-a; Kja: Kojic acid; Nod: nodularin; MC-YR: microcystin-YR; MC-LW: microcystin-LW; MC-LR: microcystin-LR.

**Table 3:** Scoring system for prioritisation of the quantified substances with the risk relevant parameters (detection frequency, biodegradability, bioaccumulation factor (BAF), and toxicity value).

Detection Frequency	Biodegradability *	Log BAF *	EC50 (mg/kg)	Score
<5%	Days	<2	>1000	0
5~30%	Weeks	2~3	100~1000	25
30~55%	Weeks–Months	3~4	10~100	50
55~80%	Months	4~5	1~10	75
>80%	Recalcitrant	>5	<1	100

* Biodegradability and BAF were estimated using EPI Suite software (United States Environmental Protection Agency, US EPA).

**Table 4:** Parameters used for the prioritisation of the tentatively identified compounds.

Toxin	CAS No.	Frequency %	Log Kow	Biodegradation Frame *	Log BAF *	LD50 (Mouse) mg/Kg	Effects	Ref.	Smileys
Phytotoxins									
Acetoxytropane	3423-26-5	71	1.5	Week–Months	1	1830	Diarrhoea and hypoactivity after administration of 50 and 200 mg/kg	[32]	CC(=O)OC12CCCC(N1C)CC2
Aconosine	38839-95-1	17	1.2	Months	0.5	0.27		[33]	CCN1CC2CCC(C34C2CC(C31)C5(CC(C6CC4C5C6O)OC)O)OC
Anethole	104-46-1	13	2.7	Weeks	2.31	2090	Lethal oral toxicity in rats at 2 g/kg	[34]	CC=CC1=CC=C(C=C1)OC
Alantolactone	546-43-0	29	3.47	Week–Months	2.06	1200	Carcinogenic/anticarcinogenic potential; Cytotoxic in vitro	[35]	CC1CCCC2(C1=CC3C(C2)OC(=O)C3=C)C
Ambrosin	509-93-3	17	1,03	Week–Months	0.21		NF-κβ inhibitor	[36,37]	CC1CCC2C(C3(C1C=CC3=O)C)OC(=O)C2=C
Apiole	523-80-8	38	2.7	Week–Months	2.21	4200	Acute oral LD50 in rats 3.96 g/kg, in mice 1.52 g/kg; acute dermal LD50 in rabbits > 5 g/kg	[38]	COC1=C2C(=C(C(=C1)CC=C)OC)OCO2
Arabsin	38412-44-1	13	0.76	Weeks	−0.02			[39]	CC1C2CCC3(C(CC(=O)C(C3C2OC1=O)C)O)C
Artemisic acid	80286-58-4	4	3.8	Week–Months	4.39	50	Cytotoxicity	[40]	CC1CCC(C2C1CCC(=C2)C)C(=C)C(=O)O
Aspidinol	519-40-4	13	2.6	Week–Months	1.01	50	anti-MRSA activity, with antibacterial effect. Inhibition of the formation of the ribosome	[41]	CCCC(=O)C1=C(C(=C(C=C1O)OC)C)O
Aspidospermine	466-49-9	13	3.78	Recalcitrant	1.76	46.3	Cytotoxicity against mouse NIH3T3 cells	[42]	CCC12CCCN3C1C4(CC3)C(CC2)N(C5=C4C=CC=C5OC)C(=O)C
Bisabolol oxide B	26184-88-3	21	2.5	Months	2.63	633	Skin reaction; hepatic toxicity	[43]	CC1=CCC(CC1)C2(CCC(O2)C(C)(C)O)C
Buddledin B	62346-21-8	13	2.9	Week–Months	2.97		Piscicidal activity	[44]	CC1=CCCC(=C)C2CC(C2C(C1=O)O)(C)C
Conhydrine	495-20-5	50	1.21	Months	0.39	11	Activation and then blocking of nicotinic acetylcholine receptors	[45]	CN1CCC23C4C1CC5=C2C(=C(C=C5)OC)OC3C(CC4)O
Cuscohygrine	454-14-8	29	1	Months	0	111	Autonomic nervous system blockade	[46]	CN1CCC[C@@H]1CC(=O)C[C@@H]2CCCN2C
Herniarin	531-59-9	29	1.74	Weeks	0.72	4300	Inhibition of human carbonic anhydrase with a concentration of 2.4 µM	[47]	COC1=CC2=C(C=C1)C=CC(=O)O2
Hygrine	496-49-1	29	0.5	Week–Months	−0.02	91		[48]	CC(=O)C[C@H]1CCCN1C
Hypoglycine A	156-56-9	33	-2.5	Day–Weeks	−0.05	98	Jamaican vomiting sickness; hypoglycaemia and death; encephalopathy	[49]	C=C1CC1CC(C(=O)O)N
Laudanosine	2688-77-9	25	3.7	Months	1.59	410	GABA receptors interaction glycine receptors, involved in epilepsy and other types of seizures	[50]	CN1CCC2=CC(=C(C=C2C1CC3=CC(=C(C=C3)OC)OC)OC)OC
Lupanine	550-90-3	38	1.6	Week–Months	0.65	410	Tremor, Muscle contraction and dyspnoea within mouse	[51]	C1CCN2CC3CC(C2C1)CN4C3CCCC4=O
Methyl-Jasmonate	1211-29-6	25	2.76	Weeks	1.25	5000	Anti-inflammatory activity in LPS-stimulation within mouse	[52]	CCC=CCC1C(CCC1=O)CC(=O)OC
Methylpelletierine	40199-45-9	17	0.8	Week–Months	0.05	40	Taenicide	[53,54]	CC(=O)CC1CCCCN1C
Methylpseudoconhydrine	140-55-6	46	1.5	Week–Months	0.33	250	Antinociceptive	[55]	CC(C(C1=CC=CC=C1)O)N(C)C
Norpseudopelletierine	4390-39-0	17	0.2	Weeks	0.15		Causes severe skin burns and eye damage; genotoxic in vitro + in vivo	[56]	C1CC2CC(=O)CC(C1)N2
p-Coumaric acid	7400-08-0	8	1.46	Day–Weeks	1.81	1.2	Reproductive toxicity	[57]	C1=CC(=CC=C1C=CC(=O)O)O
Ptaquilosin B	87625-62-5	33	ND	Months	0.42		Generation of carcinogenic ADN adducts	[35]	CC1CC2(C=C(C3(CC3)C(C2C1=O)(C)O)C)O
Reticuline	485-19-8	0	3	Months	0.61	56	Ptosis, somnolence, convulsions.	[36]	CN1CCC2=CC(=C(C=C2C1CC3=CC(=C(C=C3)OC)O)O)OC
Retronecine	480-85-3	71	-0.56	Weeks	−0.04	634	Carcinogenic, pulmonary oedema, blood lymphoma, convulsions	[38]	C1CN2CC=C(C2C1O)CO
Swainsonine	72741-87-8	17	-1.3	Weeks	−0.05	0.35	Locoweed intoxication; It is a potent inhibitor of Golgi alpha-mannosidase II	[58]	C1CC(C2C(C(CN2C1)O)O)O
Tetrahydro-cannabivarin	31262-37-0	21	5.76	Months	3.06	3	Neurotoxicity	[59]	CCCC1=CC(=C2C3C=C(CCC3C(OC2=C1)(C)C)C)O
Tetraneurin A	22621-72-3	29	0.6	Week–Months	−0.04	42	Antiviral activity; Ear thickness in rats; dermatitis	[60]	CC(=O)OCC1CCC2C(C3(C1(CCC3=O)O)C)OC(=O)C2=C
Trachelanthamine	14140-18-2	0	1.4	Week–Months	0.69	1500	Somnolence, tremor, muscle weakness	[61]	CC(C)C(C(C)O)(C(=O)OCC1CCN2C1CCC2)O
Tussilagine	80151-77-5	8	0.6	Week–Months	−0.04	28.8	Carcinogenic in vivo	[43,62]	CC1(CN2CCCC2C1C(=O)OC)O
Umbelliferone	93-35-6	21	1,58	Weeks	0.4	10000	Inhibition of human carbonic anhydrase 9 catalytic domain	[63]	C1=CC(=CC2=C1C=CC(=O)O2)O
Xanthotoxol	2009-24-7	29	1.16	Weeks	0.22	480	Inhibitors of Secretory Acid Sphingomyelinase (S-ASM);	[64]	C1=CC(=O)OC2=C(C3=C(C=CO3)C=C21)O
**Mycotoxins**									
Aflatoxin B_1_	1162-65-8	13	1.45	Week–Months	0.1	3.2	Carcinogenic, terathogenic	[65]	COC1=C2C3=C(C(=O)CC3)C(=O)OC2=C4C5C=COC5OC4=C1
Aflatoxin B_2_	7220-81-7	25	0.855	Week–Months	0.18	100	Carcinogenic, terathogenic; hepatotoxic	[66]	COC1=C2C3=C(C(=O)CC3)C(=O)OC2=C4C5CCOC5OC4=C1
Alpha-Zearalenol	36455-72-8	29	4	Weeks	1.41	0.010	Chronic toxicity and carcinogenic	[67]	CC1CCCC(CCCC=CC2=C(C(=CC(=C2)O)O)C(=O)O1)O
Aspergillic acid	2152-59-2	13	1.7	Week–Months	0.8	100	Antibiotic substance; animal toxicity	[49,68]	CCC(C)C1=CN=C(C(=O)N1O)CC(C)C
Averufin	14016-29-6	17	3	Months	1.09	20.64	Inhibition of deaminase	[69]	CC12CCCC(O1)C3=C(O2)C=C4C(=C3O)C(=O)C5=C(C4=O)C=C(C=C5O)O
Kojic Acid	501-30-4	8	-0,64	Weeks	−0.05	23.8	Inhibition of human recombinant DAAO	[70]	C1=C(OC=C(C1=O)O)CO
Azelaic acid	19619-43-3	13	1.55	Day–Weeks	0.64	5	Irritant	[71]	C(CCCC(=O)O)CCCC(=O)O
Barnol	2151-18-0	0	2.26	Week–Months	0.79			[56,62]	CCC1=C(C(=C(C(=C1C)O)O)O)C
**Cyanotoxins**									
Anatoxin-a	64285-06-9	17	0.8	Weeks	0.36	420	Neurotoxicity; muscular fasciculation, respiratory paralysis.	[72]	CC(=O)C1=CCCC2CCC1N2
MC-LR	101043-37-2	8	-1.2	Recalcitrant	−0.01	5	Hepatotoxicity; visual disturbance, respiratory irritation; vomiting, and muscle weakness	[73]	CC1C(NC(=O)C(NC(=O)C(C(NC(=O)C(NC(=O)C(NC(=O)C(=C)N(C(=O)CCC(NC1=O) C(=O)O)C)C)CC(C)C)C(=O)O)C)CCCN=C(N)N)C=CC(=CC(C)C(CC2=CC=CC=C2)OC)C
MC-LW	157622-02-1	8	5.2	Recalcitrant	0.81	0.25-0.33	Hepatotoxicity; visual disturbance, respiratory irritation; vomiting, and muscle weakness	[74]	CC1C(NC(=O)C(NC(=O)C(C(NC(=O)C(NC(=O)C(NC(=O)C(=C)N(C(=O)CCC(NC1=O)C(=O) O)C)C)CC(C)C)C(=O)O)C)CC2=CNC3=CC=CC=C32)C=CC(=CC(C)C(CC4=CC=CC=C4)OC)C
MC-YR	101064-48-6	8	-0.2	Recalcitrant	−0.02	40	Hepatotoxicity; visual disturbance, respiratory irritation; vomiting, and muscle weakness	[75]	CC1C(NC(=O)C(NC(=O)C(C(NC(=O)C(NC(=O)C(NC(=O)C(=C)N(C(=O)CCC(NC1=O)C (=O)O)C)C)CC2=CC=C(C=C2)O)C(=O)O)C)CCCN=C(N)N)C=CC(=CC(C)C(CC3=CC=CC=C3)OC)C
Nodularin	118399-22-7	4	1.7	Months	−0.04	0.060	Hepatotoxicity; visual disturbance, respiratory irritation; vomiting, and muscle weakness	[76]	CC=C1C(=O)NC(C(C(=O)NC(C(=O)NC(C(C(=O)NC(CCC(=O)N1C)C(=O)O)C)C=CC(=CC(C)C (CC2=CC=CC=C2)OC)C)CCCN=C(N)N)C)C(=O)O

* Biodegradability and BAF were estimated using EPI Suite software (United States Environmental Protection Agency, US EPA).

**Table 5:** Prioritisation for ranking the substances detected in the Ter River.

Ranking	Tentatively Identified Substance
325	Tetrahydrocannabivarin
325	MC-LW
300	Aconosine
300	MC-LR
275	MC-YR
275	Nodularin
250	Aflatoxin B1
250	Alpha-Zearalenol
225	Ptaquilosin B
225	Retronecine
225	Tussilagine
225	Aflatoxin B2
200	Aspidospermine
175	Artemisic acid
175	Conhydrine
175	Anatoxin-a
150	Bisabolol oxide B
150	Swainsonine
150	Averufin
125	Acetoxytropane
125	Apiole
125	Aspidinol
125	Cuscohygrine
125	Hygrine
125	Laudanosine
125	Lupanine
125	Methylpelletierine
125	Methylpseudoconhydrine
125	Reticuline
125	Tetraneurin A
125	Aspergillic acid
100	Alantolactone
100	Buddledin B
100	Hypoglycine A
100	p-Coumaric acid
100	Kojic Acid
100	Azelaic acid
75	Anethole
75	Ambrosin
75	Xanthotoxol
50	Arabsin
50	Herniarin
50	Methyl-Jasmonate
50	Norpseudopelletierine
50	Trachelanthamine
50	Umbelliferone
50	Barnol

## Appendix A

**Table A1:** List of the natural toxin standards employed for the confirmation.

Toxin	Toxic Group	Chemical Formula	Exact Mass	Purity (%)	Supplied by
Microcystin LA	Cyanotoxin	C46H67N7O12	909.4847	>95	Cyano (Cyanobiotech GmbH, Berlin, Germany)
Microcystin LF	Cyanotoxin	C52H71N7O12	985.5160	>95	Cyano (Cyanobiotech GmbH, Berlin, Germany)
Microcystin LR	Cyanotoxin	C49H74N10O12	994.5488	>95	Cyano (Cyanobiotech GmbH, Berlin, Germany)
Microcystin LY	Cyanotoxin	C52H71N7O13	1001.5109	>95	Cyano (Cyanobiotech GmbH, Berlin, Germany)
Microcystin LW	Cyanotoxin	C54H72N8O12	1024.5269	>95	Cyano (Cyanobiotech GmbH, Berlin, Germany)
Microcystin YR	Cyanotoxin	C52H72N10O13	1044.5353	>95	Cyano (Cyanobiotech GmbH, Berlin, Germany)
Nodularin	Cyanotoxin	C41H60N8O10	824.4432	>95	Cyano (Cyanobiotech GmbH, Berlin, Germany)
Anatoxin-a	Cyanotoxin	C10H15NO	165.2320	>98	Santa Cruz Biotechnology (Dallas, TX, USA)
Cylindrospermopsin	Cyanotoxin	C15H21N5O7S	399.1219	99	BOCSci (BOC Sciences, Ramsey Road Shirley, NY, USA)
Aflatoxin B1	Mycotoxin	C17H12O6	312.0632	>98	Merck (Darmstadt, Germany)
Ochratoxin-A	Mycotoxin	C20H18ClNO6	403.0823	>98	Merck (Darmstadt, Germany)
Baicalein	Phytotoxin	C15H10O5	270.0528	98	Merck (Darmstadt, Germany)
Genistein	Phytotoxin	C15H10O5	270.0528	>98	Merck (Darmstadt, Germany)
Amygdalin	Phytotoxin	C20H27NO11	457.158	>99	Merck (Darmstadt, Germany)
Scopolamine	Phytotoxin	C17H21NO4	303.147	>98	Merck (Darmstadt, Germany)
Cinchonine	Phytotoxin	C19H22N2O	294.1732	>98	Merck (Darmstadt, Germany)
Atropine	Phytotoxin	C17H23NO3	289.1682	>99	Merck (Darmstadt, Germany)
Kojic Acid	Mycotoxin	C6H6O4	142.0274	>98	Merck (Darmstadt, Germany)
b-Asarone	Phytotoxin	C12H16O3	208.1099	70	Merck (Darmstadt, Germany)
p-Coumaric acid	Phytotoxin	C9H8O3	164.0471	>98	Merck (Darmstadt, Germany)
Abietic acid	Phytotoxin	C20H30O2	302.2256	>95	Merck (Darmstadt, Germany)
7-Ethoxyoumarin	Phytotoxin	C11H10O3	190.0634	≥97%	Merck (Darmstadt, Germany)
7-Metoxycoumarin	Phytotoxin	C10H8O3	176.0479	>98	Merck (Darmstadt, Germany)
Arbutin	Phytotoxin	C12H16O7	272.0986	>98	Merck (Darmstadt, Germany)
Umbelliferone	Phytotoxin	C9H6O3	162.0327	>99	Merck (Darmstadt, Germany)
Thujone	Phytotoxin	C10H16O	152.1235	>99	Merck (Darmstadt, Germany)
Cotinine	Phytotoxin	C10H12N2O	176.0956	>99	Merck (Darmstadt, Germany)

**Table A2:** Inclusion list of the 100 most probable suspect compounds.

Mass [M + H]^+^	Formula [M]	CE	Toxin and Possible Isomers
239.1542	C16H18N2	35	(−)-Agroclavine
180.1019	C10H13NO2	35	(−)-Salsolinol, Fusaric acid
398.0961	C18H24BrNO4	35	(−)-Scopolamin bromide
128.1433	C8H17N	35	(+)-Coniine
142.1226	C8H15NO	35	(+)-Hygrine
249.1961	C15H24N2O	35	(+)-Lupanine
333.2060	C20 H28 O4	35	20-Deoxyingenol
184.1332	C10 H17 N O2	35	3-Acetoxytropane
197.1536	C12H20O2	35	3-Thujyl acetate
646.3221	C34H47NO11	35	Aconitine
313.0706	C17 H12 O6	70	Aflatoxin B_1_
315.0863	C17 H14 O6	35	Aflatoxin B_2_
329.065	C17 H12 O7	35	Aflatoxin G_1_
331.0812	C17H14O7	35	Aflatoxin G_2_
502.2951	C32H39NO4	35	Aflatrem
159.0513	C4 H6 N4 O3	35	Allantoin
924.4951	C47H73NO17	35	Amphotericin Bh
458.1656	C20H27NO11	60	Amygdalin
456.1511	C20H27NO11	35	Amygdalin negative
166.1226	C10 H15 N O	45	Anatoxin-A
187.03897	C11H6O3	35	Angelicin (Isopsoralen)
504.343	C28H45N3O5	35	Antillatoxin
624.3755	C34H49N5O6	35	Apicidin
271.0601	C15H10O5	35	Apigenin
283.1540	C15H22O5	35	Artemisinin
189.1121	C9 H16 O4	35	Aspionene
290.1751	C17H23NO3	50	Atropine
369.0968	C20H16O7	35	Averufin
321.1696	C18H24O5	35	a-Zearalenol
261.1597	C15H20N2O2	35	Baptifoline
784.4167	C45H57N3O9	35	Beauvericin
641.2891	C34H44N2O8S	35	Belladonnine
209.1172	C12H16O3	50	beta-Asarone
285.0757	C16H12O5	35	Biochanin A (BIO)
438.2638	C27H35NO4	35	b-Paxitriol
281.1747	C16 H24 O4	35	Brefeldin A
235.1692	C15 H22 O2	35	Buddledin B
317.2111	C20H28O3	35	Cafestol
195.0876	C8H10N4O2	35	Caffeine
153.1273	C10H16O	35	Carveol
261.1849	C17H24O2	35	Cicudiol
259.1692	C17 H22 O2	35	Cicutoxin
1111.5836	C60H86O19	35	Ciguatoxin
295.1804	C19H22N2O	35	Cinchonine
279.0863	C14H14O6	35	Citreoisocoumarin
403.2115	C23H30O6	35	Citreoviridin
400.1754	C22H25NO6	35	Colchicine
144.1382	C8H17NO	35	Conhydrine
127.0389	C6H6O3	35	Coumarin
300.2169	C16 H29 N O4	35	Curassavine
225.1961	C13H24N2O	35	Cuscohygrine
416.1234	C15H21N5O7S	45	Cylindrospermopsin
255.0651	C15H10O4	35	Daidzein (DAI)
417.1180	C21H20O9	35	Daidzin
589.1915	C29H32O13	35	Dalbin
427.1387	C23H22O8	35	Dalbinol
249.1485	C15H20O3	35	Damsin
291.1227	C16H18O5	35	Dehydrocurvularin
355.1176	C20H18O6	35	Deoxynivalenol
411.1074	C22H18O8	35	Desertorin A
367.1751	C19H26O7	35	Diacetoxyscirpenol
765.4419	C41H64O13	35	Digitoxin
415.3206	C27H42O3	35	Diosgenin
295.1903	C17 H26 O4	50	Embelin
271.0601	C15H10O5	35	Emodin
1095.5662	C60H74N10O10	35	Ergoclavin
350.1598	C18H23NO6	35	Erucifoline
269.0808	C16H12O4	35	Formononetin (FOR)
209.0444	C10H8O5	35	Fraxetin
271.0601	C15H10O5	50	Genistein or baicalein
155.1430	C10H18O	35	Geraniol
781.4368	C41H64O14	35	Gitoxin
156.1019	C8 H13 N O2	35	Heliotridine
304.1543	C17H21NO4	35	Hyoscine
143.0338	C6H6O4	35	Kojic acid
541.3887	C34 H52 O5	35	Lantadene D
358.2012	C21 H27 N O4	35	Laudanosine
910.4920	C46H67N7O12	35	MC-LA
995.5560	C49H74N10O12	35	MC-LR
1025.5344	C54H72N8O12	35	MC-LW
1045.5353	C52H72N10O13	35	MC-YR
192.0781	C11H12O3	35	Myristicin
825.4505	C41 H60 N8 O10	35	Nodularin
128.1069	C7H13NO	35	Norhygrine
152.0566	C5H5N5O	35	Nostocine
404.0895	C20H18ClNO6	70	Ochratoxin-a
215.1277	C11H18O4	35	Pestalotin
165.0658	C8H8N2O2	35	Ricinine
194.1175	C11H15NO2	35	Salsoline
868.5053	C45H73NO15	35	Solanine
746.4837	C42H67NO10	35	Spirolide
183.0288	C8H6O5	35	Stipitatic acid
174.11247	C8H15NO3	35	Swainsonine
153.1273	C10H16O	35	Thujone
115.0389	C5H6O3	35	Tulipalin B
163.0389	C9H6O3	35	Umbelliferone
355.2380	C22 H30 N2 O2	35	Vincaminorein (Aspidospermine)
203.0338	C11 H6 O4	35	Xanthotoxol

**Table A3:** Calibration curve parameters for the quantification of the confirmed compounds.

Toxins	Molecular Formula	[M+H]+	Recovery%	RSD%	LOD µg/L	LOQ µg/L	R^2^
**Ana**	C_10_H_15_NO	166.1234	84	8.0	0.2	0.5	0.989
**AflB_1_**	C_17_H_12_O_6_	416.1242	86	9.9	0.2	0.7	0.999
**MC-LR**	C_49_H_74_N_10_O_12_	995.5568	78	3.3	0.2	0.5	0.995
**MC-LW**	C_54_H_72_N_8_O_12_	1025.5342	55	5.8	0.1	0.5	0.991
**Nod**	C_41_H_60_N_8_O_10_	825.4512	94	16.2	0.2	0.8	0.992
**MC-YR**	C_54_H_72_N_8_O_12_	1045.5361	84	16.9	0.4	1.5	0.943
**Kja**	C_12_H_16_O_3_	208.1093	85	6.4	0.02	0.08	0.990
**7-methoxycoumarin**	C_10_H_8_O_3_	177.0546	82	7	0.002	0.007	0.999
**Umbelliferone**	C_9_H_6_O_3_	163.0389	79	11.2	0.009	0.03	0.998
